# Navigating Barriers and Challenges: A Narrative Review of the Experiences of Autistic People and Their Families in Latin America

**DOI:** 10.7759/cureus.113505

**Published:** 2026-07-28

**Authors:** Alfredo de Jesús Garza Guerra, Elisa del Rocio Mata Cortes, Gabriela Hilian Adame Rocha, Stefan Mauricio Fernández Zambrano

**Affiliations:** 1 Psychiatry, Hospital Universitario Dr. José Eleuterio Gonzalez, Monterrey, MEX; 2 Psychiatry, Jalisco Institute of Mental Health and Addictions, University of Guadalajara, Guadalajara, MEX

**Keywords:** autism, autistic people, educational inclusion, family experiences, health services, latin america, social protection

## Abstract

Research on autism in Latin America has grown substantially over the past two decades; however, evidence regarding the experiences of autistic people and their families remains fragmented across countries and disciplines. This narrative review aimed to synthesize the available evidence on the experiences of autistic people and their families while navigating health, education, and social protection systems across Latin America. A literature search was conducted in PubMed for studies published between January 2000 and July 2026. The search combined the terms "autism spectrum disorder," "autism," and "autistic" with the names and demonyms of all countries in Latin America and the Caribbean, as well as the regional terms Latin America and Central America. Studies addressing pathways to autism identification, access to health services, socioeconomic inequalities, family experiences, educational inclusion, professional training, and current regional challenges were included.

A total of 75 studies met the inclusion criteria and were synthesized narratively. Across the region, families consistently recognized developmental differences during the first years of life; however, pathways to autism identification were frequently prolonged by fragmented health systems, shortages of trained professionals, and limited access to specialized services. Socioeconomic and geographic inequalities further restricted access to assessment, supports, and ongoing services. Families played a central role in coordinating care, navigating complex systems, and advocating for the rights of autistic people. Educational inclusion remained limited by insufficient resources, inadequate teacher preparation, and inconsistent implementation of inclusive policies. The available evidence was concentrated in a small number of countries, highlighting important gaps in regional knowledge. Overall, autistic people and their families continue to encounter substantial barriers while navigating health, education, and social protection systems across Latin America. Strengthening early identification, expanding professional training, improving intersectoral coordination, and increasing research capacity across underrepresented countries are essential priorities for advancing equitable, coordinated, and inclusive supports throughout the region.

## Introduction and background

Autistic people and their families interact throughout the life course with health, education, social protection, and community support systems that influence access to autism identification, specialized services, inclusive education, and social participation. In Latin America, these experiences occur within contexts marked by socioeconomic inequalities, fragmented systems, unequal distribution of specialized resources, and considerable variation in the implementation of relevant public policies [1-5]. Although autism research in Latin America has grown steadily over the past two decades, the available evidence remains dispersed across countries and disciplines [1,5-9]. This fragmentation limits the identification of shared regional patterns, differences between national contexts, and persistent gaps in knowledge.

Collectively, studies conducted across the region indicate that these experiences are shaped by structural barriers, including delays in autism identification, limited availability of trained professionals, geographic and socioeconomic inequalities, fragmented service delivery, insufficient coordination across health, education, and social protection sectors, and restricted access to timely and continuous supports [4,5,9-16]. These barriers not only limit access to services and supports but also influence social participation, educational inclusion, family well-being, and the realization of the rights of autistic people.

This narrative review adds to existing knowledge by bringing together findings from different countries, disciplines, and service systems within a regional and intersectoral synthesis. By identifying recurring barriers, underrepresented countries, and gaps in the available evidence, it may provide a foundation for future multicountry and contextually appropriate research while informing efforts to improve autism identification, support, and social participation in Latin America. Accordingly, this review aimed to synthesize the available evidence on the experiences of autistic people and their families while navigating health, education, and social protection systems across Latin America.

## Review

Methods

Study Design

A narrative literature review was conducted to synthesize evidence on the experiences of autistic people and their families while interacting with health, education, social protection, and support systems across Latin America. A narrative approach was selected because the available literature encompasses heterogeneous study designs, populations, national contexts, service systems, and thematic areas that are not readily suited to quantitative pooling. This approach allowed evidence from different disciplines to be integrated and recurring regional patterns, contextual differences, and knowledge gaps to be identified.

Search Strategy

A literature search was conducted in PubMed for studies published between January 2000 and July 2026. The search combined the terms “autism spectrum disorder,” “autism,” and “autistic” with the name and corresponding demonym of each country and territory in Latin America and the Caribbean. Additional independent searches were conducted using the regional terms “Latin America” and “Central America” to identify multicountry and regional studies. Equivalent search combinations were applied throughout the review. For example: (“autism spectrum disorder” OR autism OR autistic) AND Brazil. The same strategy was replicated for each country, territory, demonym, and regional term.

The search was restricted to PubMed. Although this database provides broad coverage of biomedical, health, and behavioral research, relevant studies indexed exclusively in other regional or international databases, or available as gray literature, may not have been identified. Consequently, this review should be interpreted as a synthesis of the regional literature captured through PubMed.

Eligibility Criteria

Studies were included if they examined the experiences of autistic people or their families in Latin America in relation to pathways to autism identification, access to or organization of health services, socioeconomic inequalities, family and caregiving experiences, educational inclusion, professional knowledge and training, or broader challenges associated with autism-related support. Studies focusing exclusively on genetic, neurobiological, pharmacological, biomedical, or experimental aspects of autism were excluded. Case reports, editorials, letters to the editor, conference abstracts, and publications that did not address the objectives of the review were also excluded.

Study Selection and Reviewer Involvement

**Figure 1:**
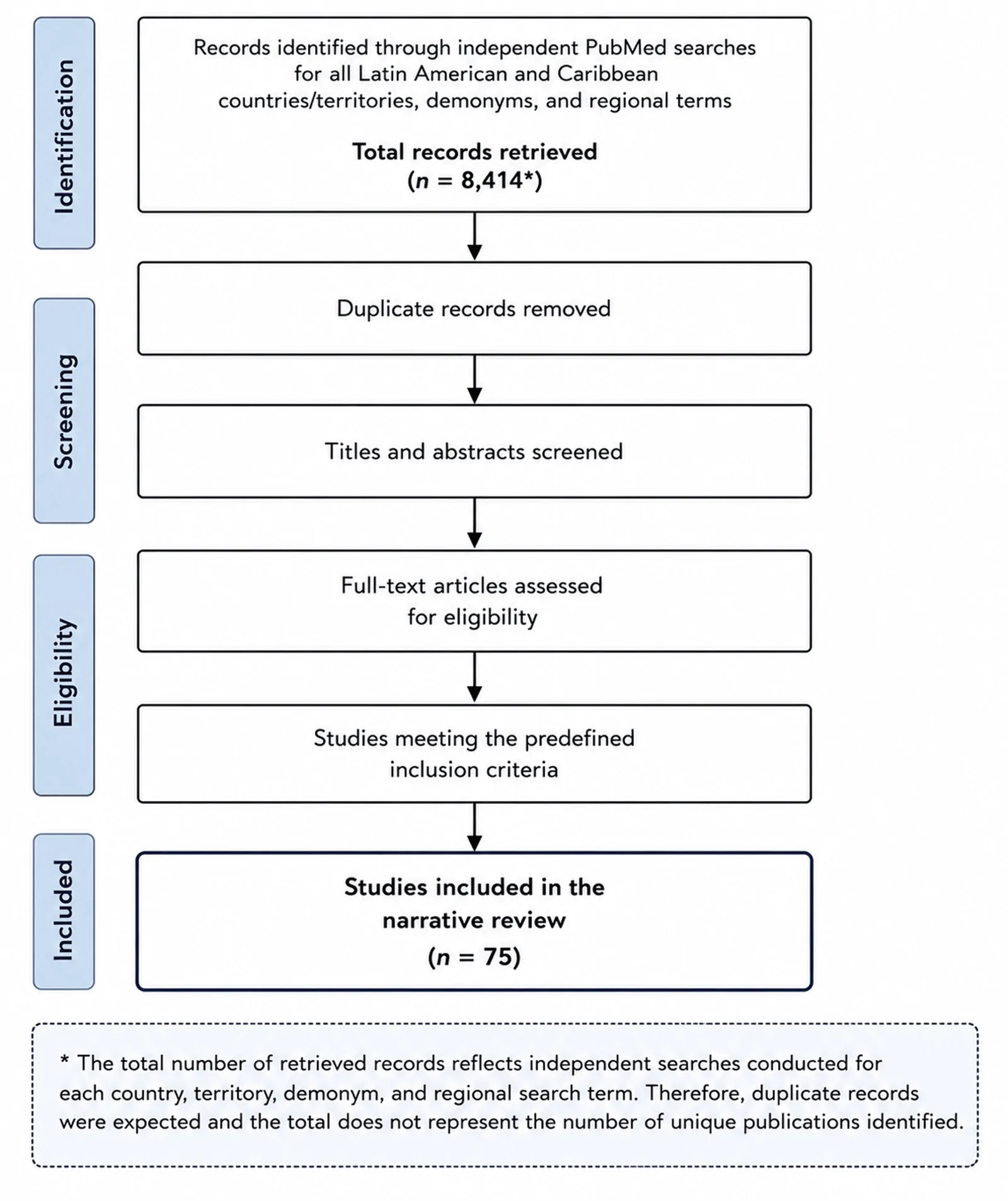
Flowchart depicting the study-selection process

The independent searches yielded a cumulative total of 8,414 records, including overlapping results from searches by country, territory, demonym, and regional term. After duplicates were removed manually, all authors jointly screened titles and abstracts and assessed potentially eligible full-text articles according to the predefined criteria. Final inclusion decisions were reached by consensus. The study-selection process is summarized in Figure 1. Following screening and full-text assessment, 75 studies were included in the narrative synthesis. Because individual publications could appear in multiple search combinations, the initial total represents cumulative search results rather than the number of unique publications.

Data Extraction and Synthesis

The authors jointly extracted data on country, study objectives, design, participants, service context, and findings relevant to the review. The evidence was synthesized narratively by comparing findings across studies to identify recurring patterns, contextual differences, and regional knowledge gaps.

Findings were organized into the following thematic categories: pathways to autism identification; access to and organization of health services; socioeconomic inequalities; family experiences and support needs; education and educational inclusion; training and challenges faced by health professionals; and current priorities for autism support across Latin America. Studies addressing more than one topic could contribute to multiple categories. Given the heterogeneity of study designs, populations, outcomes, and national contexts, no meta-analysis or other quantitative synthesis was undertaken. The geographic distribution of the included studies is presented in Table 1.

**Table 1:** Geographical distribution of studies included in the narrative review The search strategy included independent PubMed searches for all countries and territories in Latin America and the Caribbean using country names, demonyms, and the regional terms Latin America and Central America. Only countries or regions contributing studies that met the inclusion criteria are presented

Country	Number of articles
Brazil	21
Chile	9
Mexico	7
Argentina	6
Ecuador	5
Colombia	4
Venezuela	3
Uruguay	3
Costa Rica	3
Puerto Rico	2
Paraguay	1
Peru	1
Dominican Republic	1
Latin America/Central America	9
Total	75

Methodological Quality of the Evidence

Given the narrative approach and heterogeneity of the included study designs, no formal risk-of-bias assessment or standardized methodological quality assessment was performed. However, the design, sample characteristics, scope, and contextual limitations of individual studies were considered when interpreting the evidence.

Findings: thematic narrative synthesis

The findings were organized into six interrelated thematic areas. Although presented separately for clarity, barriers involving autism identification, services, socioeconomic conditions, family support, education, and professional practice frequently intersected. The evidence also varied substantially in design, population, and national context and was concentrated in a limited number of countries. The findings should therefore be interpreted as recurring patterns identified across the available literature.

Pathways to Autism Identification and Access to Health Services

Autism identification often represents the first contact of autistic people and their families with health and education systems. In the countries represented, this process encompassed recognition of developmental differences, help-seeking, professional consultation, and access to specialized assessment, with its duration shaped by individual, family, social, and structural factors [8,17,18].

Across most studies, families first expressed concerns between 18 and 24 months of age. Language-related differences were the most frequent reason for seeking support, followed by differences in social interaction, response to their name, play, sensory processing, and behavioral regulation [6,10-12,16,19-23]. Despite this early recognition, the reported mean age of autism identification generally ranged from approximately 40 to 58 months, and the interval between initial concerns and specialized assessment varied from approximately 13 months to three years. Although some recent studies suggested a trend toward earlier identification, delays remained common across the settings examined [6,9-12,16,19,23-27].

Families described repeated referrals, poor coordination, unclear care pathways, and uncertainty about where to obtain an assessment. In some studies, they consulted as many as 11 professionals before completing the process. Initial concerns were also sometimes minimized or attributed to typical developmental variation [16-18,22,28,29].

System-level constraints included limited autism expertise and training, insufficient developmental surveillance and screening, the urban concentration of specialized services, weak referral systems, and limited availability of culturally appropriate and validated assessment tools [5,7-10,29-33]. Later identification was associated with lower socioeconomic status, rural residence, lower caregiver educational attainment, and autistic characteristics that did not align with stereotypical presentations [11,25,27,29,31,33-35]. Autism may therefore remain underidentified among populations experiencing greater structural barriers; however, its extent cannot be determined from the available studies [29,31,33,35].

Concerns also extended to the quality of assessment. Families reported uncertainty, brief or incomplete evaluations, inconsistent use of standardized instruments, and insufficient information about findings and available supports [17,18,36]. Assessment should therefore provide information that guides individualized support according to each person’s functioning, participation, strengths, and support needs [37]. Challenges frequently persisted after identification. Although several countries have introduced legislative and policy frameworks intended to protect the right to health and improve access to autism-related services, gaps remained between policy and implementation. Insufficient funding, system fragmentation, and regional inequalities continued to affect the availability and continuity of support [2-5,8,13].

Specialized multidisciplinary services were often concentrated in private institutions or non-governmental organizations, while public-sector provision remained limited in several settings, restricting coordinated and continuous service delivery [4,6,7,15,17,38,39]. Families consequently assembled their own support networks, sometimes without coordination or continuity across services [15,18]. Other obstacles included long waiting periods, high costs, inadequate public or insurance coverage, restricted access to developmental assessments, and insufficient information about available resources [1,4,28,40-42]. Long intervals between appointments, financial interruptions, delayed referrals, and considerable travel distances further disrupted support [15,18,26].

Within-country geographic disparities were also evident. Economically developed areas with greater concentrations of specialized professionals generally reported higher service availability and autism identification rates than resource-limited areas [31,33,43]. This pattern appears to reflect unequal healthcare organization and resource distribution rather than established geographic differences in autism prevalence [2,33,43,44]. Limited national registries and integrated information systems further reduced the capacity to estimate needs, evaluate service provision, and inform planning and resource allocation [4,44-46]. Overall, the evidence indicates that barriers at different stages, from initial recognition to continuing support, are interconnected. However, heterogeneity in study design, population, and healthcare organization precluded direct comparisons.

Socioeconomic Inequalities in Access to Supports

Socioeconomic conditions influenced access to autism identification, healthcare, and continuing support in the countries examined. Families reported direct costs related to assessment and services, as well as transportation expenses, reduced household income, and productivity losses [1,14,40,42,47,48]. Broader economic conditions, including inflation, employment instability, and fragmented health systems, could also interrupt support when families were unable to cover these costs [49-50].

In resource-limited settings, basic household needs were sometimes prioritized over specialized assessments and support services. This contributed to delayed or discontinued support, particularly in rural and underserved areas [4,5,7,9,31,33,44,51]. Household income and health insurance coverage also influenced pathways to support. Families with greater financial resources were more likely to access private services, experience shorter waiting periods, and obtain specialized assessments. In contrast, those dependent on publicly funded systems frequently experienced longer pathways and more limited service availability [14,15,17,22,52,53].

These findings consistently indicate an association between socioeconomic resources and access to services. However, differences in national financing arrangements, insurance systems, and measures of socioeconomic status limit direct comparisons among countries. The findings should therefore be understood within the economic and institutional contexts in which each study was conducted.

Family Experiences, Caregiving, and Support Needs

Families were consistently described as a principal source of support for autistic people, participating in the recognition of developmental differences, help-seeking, service coordination, and rights advocacy. In many settings, they also acted as intermediaries between health, education, and social protection systems [3,41,47,49,54]. Mothers were often the first family members to recognize developmental differences and initiate help-seeking. Their concerns, however, were sometimes minimized by relatives or healthcare professionals, contributing to uncertainty and delayed access to assessment and support [6,9,10,17,40,50].

Family understandings of autism were shaped by cultural, social, and historical contexts. Some studies described limited knowledge before identification and explanations based on stereotypes, parenting practices, religious beliefs, or spiritual interpretations. Such beliefs could contribute to stigma, delayed help-seeking, guilt, shame, or self-blame [2,53,55-57]. Following identification, families frequently reported uncertainty, sadness, fear, guilt, and concern about the future. Over time, many developed coping strategies, increased their understanding of autism, and established networks that supported adaptation and participation in decision-making. Spirituality and social support were also identified as important resources in several contexts [17,28,47-49,54,58].

Caregiving responsibilities fell predominantly on mothers, who frequently reorganized employment, family life, and social participation around their children’s support needs. This unequal distribution was associated with emotional, physical, and financial strain, although some studies described increasing paternal involvement [28,47,48,50,54,58,59]. Limited service availability further increased families’ responsibilities for obtaining information, coordinating support, and advocating for their rights. Concerns about autonomy, employment, housing, and continuity of support during adulthood were also prominent [5,8,41,49,60-65].

Extended family and peer support groups could strengthen resilience by offering practical assistance, shared experience, and access to information. However, extended-family involvement was not uniformly supportive, as some relatives minimized or stigmatized autism [47-49,54,58,61,62]. Overall, the literature portrays families as compensating for limitations within formal health, education, and social protection systems [45,47,49,54,61,62,66].

Education and Educational Inclusion

Education was a major setting for participation and a central concern for families. Although several Latin American countries have adopted inclusive education frameworks, the reviewed studies described gaps between legal recognition and implementation [1,8,40,42,64,66]. Schools could contribute to autism identification when developmental differences became more apparent after entry into formal education. In such cases, later recognition could reduce opportunities for earlier educational and developmental support. Some studies also suggested that students whose characteristics were less consistent with stereotypical presentations received support later than their peers [16,31,35,52,63,64].

Implementation of inclusive education was constrained by limited infrastructure and resources, insufficient teacher preparation, and unclear procedures for providing classroom support. Some institutions reportedly refused enrollment or required the continuous presence of an aide or support person, thereby creating additional barriers to participation [5,8,42,63,64,66]. Other reported challenges included limited individualized support and reasonable accommodations, poor coordination between health and education systems, reduced attendance, negative attitudes, and bullying. Schools also experienced difficulty providing physically, socially, and sensorily accessible environments and implementing curricular adaptations that were responsive to individual strengths and support needs [1,42,57,63,64,66,67].

Educational arrangements varied according to national policy and resource availability and included mainstream schools, resource classrooms, specialized centers, residential institutions, and home-based education. This variability reflects differences in the organization and capacity of the educational systems examined and frequently increased families’ responsibility for coordinating support [4,5,30,42,45,64,66]. The evidence therefore indicates that formal access to schooling does not necessarily constitute meaningful inclusion. However, substantial variation in educational policy, terminology, infrastructure, and research methods limits direct comparisons among countries. Conclusions regarding educational inclusion should consequently be restricted to the settings represented in the available literature [1,5,42,45,64,66].

Health Professionals: Training, Practice, and Challenges in Autism Care

Health professionals play a central role in autism identification, assessment, information provision, and continuing support. Effective communication, guidance, and continuity of care can facilitate family adaptation, service engagement, and planning across the life course [47-49,54,68]. Studies identified gaps in autism-related undergraduate education and continuing professional training. These gaps affected confidence in recognizing autism, making appropriate referrals, and implementing evidence-based practices [6,8,9,20,32,38,39,65,69].

Many professionals consequently obtained autism-related knowledge through clinical experience, mentorship, self-directed learning, or continuing education rather than formal specialist preparation. Limited availability of locally validated assessment instruments and inconsistent use of standardized tools also contributed to variation in assessment practices and the recognition of co-occurring conditions [3,9,20,36-38,70-72]. Professional practice was additionally shaped by organizational constraints, including limited coordination between levels of care, short consultation times, unfamiliarity with developmental surveillance and screening tools, and uncertainty when discussing developmental concerns with families [17,20,22,32,65,66,69]. Some studies also suggested that professionals may continue to rely on narrow or stereotypical conceptions of autism, potentially reducing the recognition of diverse autistic presentations [10,17,18,36,40,55,57].

These findings support the need for improved professional education and stronger clinical pathways. Nevertheless, the available studies used different populations, instruments, and definitions of knowledge or competence. The evidence therefore demonstrates variation in training and practice but does not permit standardized comparisons of professional competence across countries.

Current Challenges and Priorities for Autism Support in Latin America

Across the reviewed studies, barriers extended beyond early identification and reflected the interaction of fragmented health systems, educational limitations, socioeconomic inequalities, and insufficient coordination among service sectors [1,2,5,45,66]. Priorities identified in the literature included strengthening developmental surveillance, referral pathways, comprehensive assessment, professional training, and access to coordinated services, particularly in underserved areas [32,38,39,66,69].

In education, the findings supported moving beyond placement toward meaningful inclusion through teacher preparation, reasonable accommodations, accessible environments, and collaboration among education, health, and social protection systems [63,64,66,67]. Family-centered approaches may help reduce caregiving strain and improve access to information, community networks, and support across the life course [49,54,61,62,73]. National registries, surveillance systems, and more reliable service data could strengthen planning, policy evaluation, and resource allocation [33,44,46,73]. Greater intersectoral collaboration and meaningful participation by autistic people, families, and representative organizations may improve the relevance and implementation of policies and services [2,45,55,66,67].

These priorities derive from evidence concentrated in a limited number of countries and should be adapted to local socioeconomic conditions, educational structures, public policies, cultures, and healthcare systems. Although some findings may be relevant to other underserved settings, they should not be assumed to be directly transferable to other parts of the world without contextual evaluation.

Discussion

This narrative review synthesized evidence on the experiences of autistic people and their families while interacting with health, education, and social protection systems in Latin America. Across the countries represented, the literature identified recurring barriers to autism identification, specialized services, educational inclusion, and coordinated support. These barriers appeared to reflect interconnected characteristics of service systems rather than isolated difficulties within individual sectors [1,2,5,45,66].

A consistent finding was the interval between families’ initial developmental concerns and access to specialized assessment. Families often recognized developmental differences during early childhood, but assessment occurred months or years later. Reported explanations included shortages of trained professionals, fragmented services, limited developmental surveillance, and inefficient referral pathways [5,7,9,17,30,32]. Similar barriers have been described internationally, but their expression in Latin America is shaped by socioeconomic inequalities, unequal distribution of specialized resources, and differences in healthcare organization [5,31,65].

Barriers identified during the assessment process frequently continued across the life course. Limited service availability, geographic disparities, financial constraints, and insufficient coordination among health, education, and social protection systems contributed to fragmented support pathways [3-5,15,45,66]. Families consequently assumed responsibilities extending beyond everyday caregiving, including locating services, coordinating providers, navigating administrative systems, and advocating for their rights. Evidence from caregiver studies and family-mediated programs similarly demonstrates the central role of families in compensating for limitations within formal systems [39,49,54,61,62,72,74].

Educational inclusion was another area in which legal recognition frequently exceeded implementation. The reviewed studies described substantial variation in teacher preparation, individualized support, reasonable accommodations, accessibility, and collaboration between education and health services. These findings suggest that school placement alone does not ensure meaningful inclusion, which also requires the resources and institutional capacity to respond to individual strengths and support needs [5,42,63,64,66].

The convergence of findings across different thematic areas increases confidence that structural and intersectoral barriers represent an important regional concern. Nevertheless, the strength of the conclusions is limited by methodological heterogeneity, differences in populations and outcomes, and variation among national service systems. Because no standardized methodological quality or risk-of-bias assessment was conducted, the relative strength of individual studies could not be formally compared.

The geographic distribution of the evidence further limits regional inference. More than 60% of the included studies originated from Brazil, Chile, Mexico, and Argentina, while many Latin American and Caribbean countries contributed little or no evidence. This concentration may reflect differences in research infrastructure, funding, academic capacity, database indexing, and publication practices rather than differences in the experiences or support needs of autistic people. Consequently, the findings do not represent the full cultural, linguistic, socioeconomic, educational, and healthcare diversity of the region [5,44,67,68,75].

Future research should expand beyond the countries that currently dominate the literature and incorporate greater methodological diversity, multicountry collaboration, standardized outcomes, and longitudinal approaches capable of examining experiences across the life course [5,31,67]. National registries, surveillance systems, and routinely collected service data could strengthen service planning, policy evaluation, and equitable resource allocation [33,44,46,73]. Greater participation by autistic people, families, and community organizations throughout the research process could also improve the relevance, contextual appropriateness, and implementation of future studies [61,62,67].

This review has several limitations. First, the search was restricted to PubMed, and relevant studies indexed exclusively in other regional or international databases or available as gray literature may not have been identified. This restriction may have introduced database coverage and publication biases and means that the review should not be considered an exhaustive synthesis of all evidence produced in Latin America. Second, the methodological heterogeneity of the included studies limited direct comparisons and precluded quantitative synthesis. Despite these limitations, the review brings together evidence from different countries, disciplines, and service sectors within a regional and intersectoral synthesis. It identifies areas of convergence, important contextual differences, and gaps that may guide future research without assuming that the available evidence is equally representative of all countries.

## Conclusions

The available evidence indicates that autistic people and their families in the Latin American settings represented in this review encounter interconnected barriers across health, education, and social protection systems. Pathways to autism identification are frequently prolonged, access to specialized services is uneven, and socioeconomic and geographic inequalities affect the timeliness and continuity of support. Families often assume a central role in coordinating services and advocating for rights, sometimes compensating for limitations within formal systems. Although regional autism research has expanded over the past two decades, the evidence remains geographically concentrated and methodologically heterogeneous. The reviewed literature supports strengthening developmental surveillance and identification pathways, professional training, intersectoral coordination, family support, and meaningful educational inclusion. However, these priorities should be adapted to each country’s cultural, socioeconomic, educational, policy, and healthcare context. Expanding research in underrepresented countries and increasing the participation of autistic people and their families will be important for developing more equitable, coordinated, and contextually appropriate systems of support.
